# Binge Drinking and Heavy Drinking Among Older Military Veterans: Applying the Theory of Intersectionality

**DOI:** 10.1093/geroni/igab046.1054

**Published:** 2021-12-17

**Authors:** Zainab Suntai, Justin McDaniel, David Albright, Julianne Wallace

**Affiliations:** 1 University of Alabama, Tuscaloosa, Alabama, United States; 2 Southern Illinois University, Carbondale, Illinois, United States

## Abstract

The post-service impact of military experiences include post-traumatic stress disorder, depression, substance misuse and several other adverse outcomes that persist well into older adulthood. As such, older military veterans are at risk of developing alcohol dependency and those with existing stressors from other identities are at the highest risk of engaging in binge drinking or heavy drinking. This study used the theory of intersectionality to examine alcohol misuse by veteran status and age, veteran status and race and veteran status and sex. Data were derived from the 2016, 2017 and 2018 Brief Risk Factor Surveillance System (BRFSS) from the Centers for Disease Control and Prevention (CDC). The BRFSS is an annual survey conducted over the phone in all 50 states and territories. Survey-weighted logistic regression models were used to examine alcohol misuse among adults aged 65+ by veteran status and the intersection between age, race, and sex. Results showed no interaction between veteran status and age, and no interaction between veteran status and sex. However, there was a significant interaction between veteran status and race, in that Black/Other race veterans were more likely to engage in both binge drinking and heavy drinking compared to White veterans, White nonveterans and nonveterans of the same race. Interventions geared towards this population should therefore engage culturally sensitive approaches that consider the historical and systemic factors that contribute to these disparities in rates of alcohol misuse among older military veterans.

